# Supplementary material for: RNF168-mediated localization of BARD1 recruits the BRCA1-PALB2 complex to DNA damage
Source: Nat Commun. 2021 Aug 18;12:5016. doi: 10.1038/s41467-021-25346-4 (PMC8373961; doi:10.1038/s41467-021-25346-4)
Supplement: Supplementary file 1 — Supplementary Information [file 41467_2021_25346_MOESM1_ESM.pdf]

SUPPLEMENTARY INFORMATION

Supplementary Figures

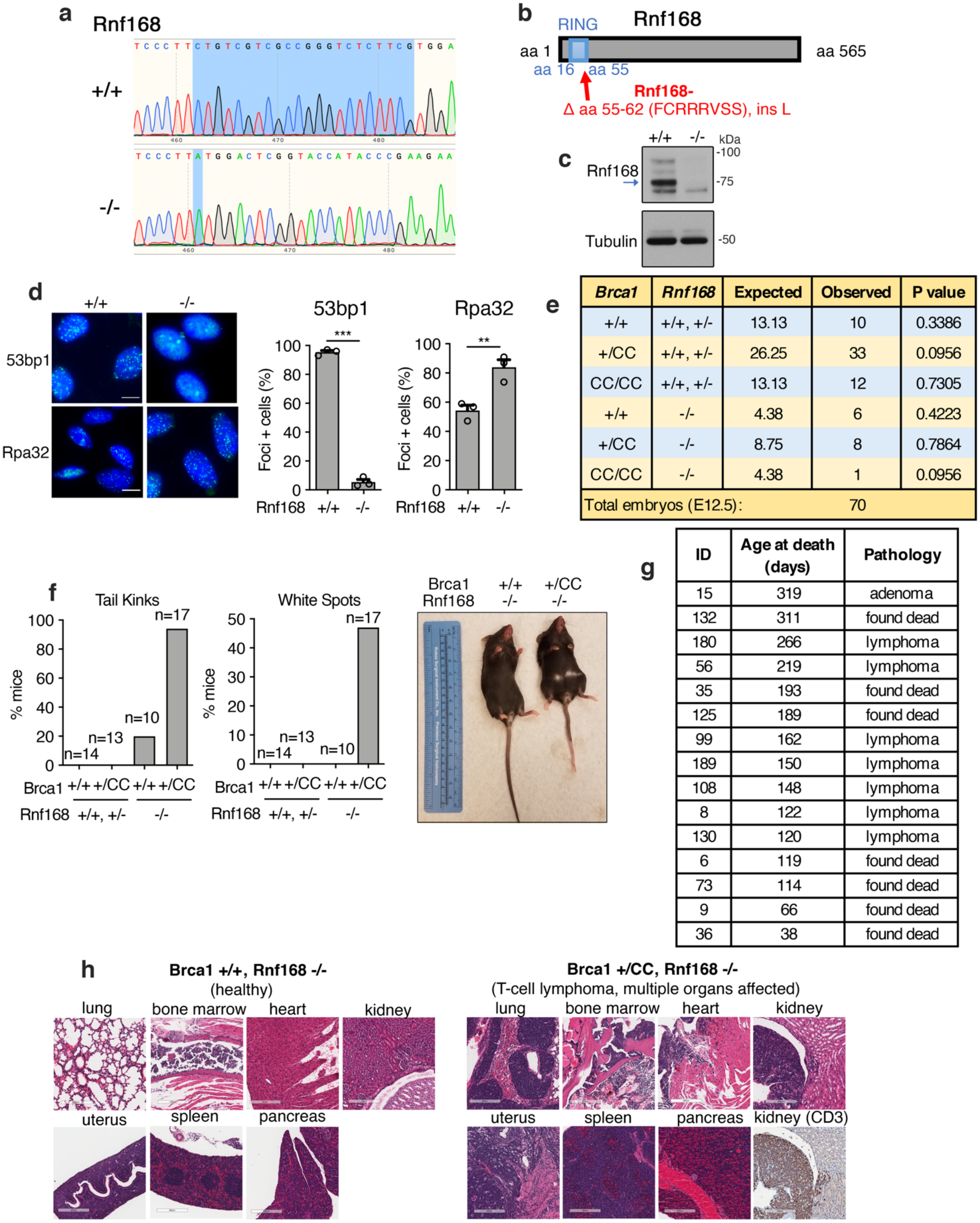

e

Brca1	*Rnf168*	Expected	Observed	P value
+/+	+/+, +/-	13.13	10	0.3386
+/CC	+/+, +/-	26.25	33	0.0956
CC/CC	+/+, +/-	13.13	12	0.7305
+/+	-/-	4.38	6	0.4223
+/CC	-/-	8.75	8	0.7864
CC/CC	-/-	4.38	1	0.0956
Total embryos (E12.5):			70	

g

ID	Age at death (days)	Pathology
15	319	adenoma
132	311	found dead
180	266	lymphoma
56	219	lymphoma
35	193	found dead
125	189	found dead
99	162	lymphoma
189	150	lymphoma
108	148	lymphoma
8	122	lymphoma
130	120	lymphoma
6	119	found dead
73	114	found dead
9	66	found dead
36	38	found dead

Supplementary Figure 1. Phenotypes associated with *Rnf168*$^{-}$ and *Brca1*^{CC} mice, related to Fig. 1.

(**A**) Electropherogram showing *Rnf168* wild-type (+/+) and mutant (-/-) alleles. The *Rnf168*$^{-}$ allele has a deletion of CTGTCGTCGCCGGGTCTCTTCG and insertion A at c.165.

(**B**) Cartoon showing the impact of the above *Rnf168*$^{-}$ mutation on the Rnf168 protein. *Rnf168*$^{-}$ generates an in-frame deletion of amino acids (aa) 55-62 plus an insertion of an L.

(**C**) Rnf168 protein expression was assessed in *Rnf168*$^{+/+}$ and *Rnf168*$^{-/-}$ MEFs using an antibody recognizing an epitope spanning aa 423-565. Despite the *Rnf168*$^{-}$ allele generating an in-frame deletion no protein was detected by Western blotting, likely from protein destabilizing effects of the deletion mutation that is proximal to the Ring domain.

(**D**) 53bp1 and Rpa32 IRIF were measured in *Rnf168*$^{+/+}$ and *Rnf168*$^{-/-}$ derived MEFs. *Left*; representative images (scale bar, 10 μm). *Right*; mean and S.E M. percentage (≥10) foci positive cells, n=3 biological replicates. *** $p < 0.001$, ** $p < 0.01$, (unpaired, two-tailed t-tests).

(**E**) Live embryos from *Brca1*$^{+/CC}$*, Rnf168*$^{+/-}$ *x Brca1*$^{+/CC}$*, Rnf168*$^{+/-}$ crosses were collected and genotyped at E12.5. The numbers of expected and observed live embryos is shown. P value are obtained from two-sided chi-square goodness of fit tests for the binomial of each genotype.

(**F**) Mice with the indicated genotypes were recorded for the number born with tail kinks and white spots and expressed as a percentage of the number of mice evaluated for each of the indicated genotypes. *Right*; photograph of representative mice.

(**G**) Table describing age and available pathology at time of death associated with *Brca1*$^{+/CC}$*, Rnf168*$^{-/-}$ mice.

(**H**) The indicated organs and tissues were H&E stained and subject to pathological inspection. Images are shown from a representative healthy *Brca1*$^{+/+}$*, Rnf168*$^{-/-}$ mouse and *Brca1*$^{+/CC}$*, Rnf168*$^{-/-}$ mouse with lymphoma (n=7, from G) detected in multiple organs (scale bars, 200 μm). CD3 immunohistochemical staining of the lymphocytic infiltration of the kidney (bottom right) indicates T-cell lymphoma.

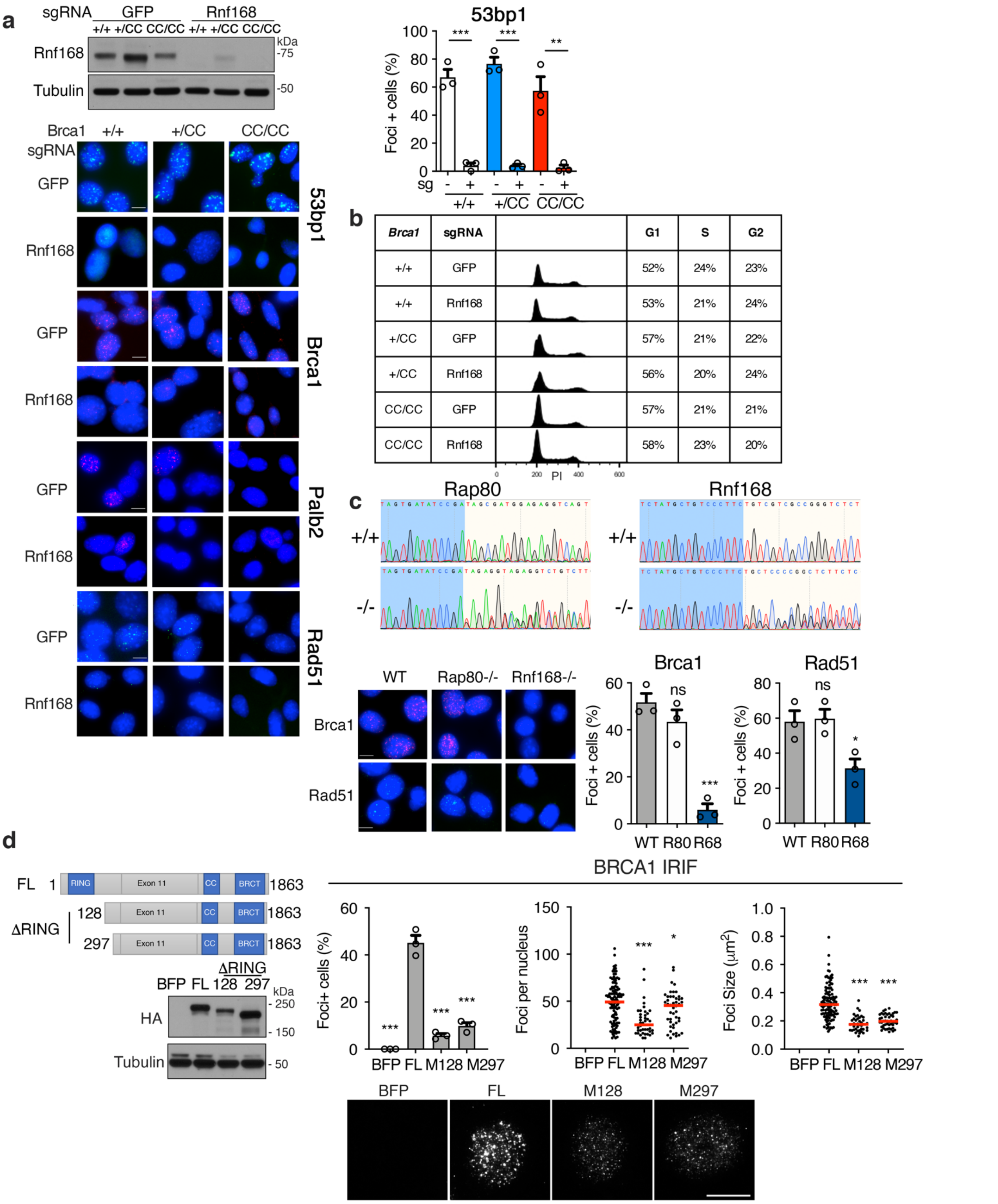

Supplementary Figure 2. Characterization of *Rnf168*⁻ and *Brca1*^{CC} MEFs, related to Fig. 1&2.

(**A**) *Brca1*$^{+/+}$, *Brca1*$^{+/CC}$, and *Brca1*$^{CC/CC}$ MEFs were incubated with lentivirus expressing sgRNA targeting *GFP* (control) or *Rnf168* and cells collected for Western blotting to confirm loss of Rnf168 expression. *Below*; representative pictures of cells from Fig. 1F (scale bar, 10 μm). 53bp1 IRIF were quantified as described in Fig.

1F and shown as mean and S.E.M for n=3 biological replicates. *** $p < 0.001$, ** $p < 0.01$, * $p < 0.05$, ^{ns} not significant (unpaired, two-tailed t-tests).

(**B**) To confirm that differences in foci formation were not caused by significant changes in cell cycle distribution, cells from A were assessed for cell cycle fractions using propidium iodide (PI) staining and flow cytometry.

(**C**) MEFs were subject to sgRNA targeting *Rnf168, Rap80* or *GFP* (WT), clones established with frameshifting mutations and assessed for Brca1 and Rad51 IRIF. *Above*; Electropherograms and frameshift mutations are shown for *Rap80*$^{-/-}$ and *Rnf168*$^{-/-}$ clones. *Below*; representative pictures (scale bar, 10 μm) and mean and S.E.M. percentage BRCA1 (≥10) foci and RAD51 (≥5) foci positive cells. MEFs n=3 biological replicates. *** $p < 0.001$, * $p < 0.05$, ^{ns} not significant compared to WT MEFs (unpaired, two-tailed t-tests).

(**D**) *Left*; cartoon showing BRCA1 protein domains and truncations with aa numbers indicated. *Below*; HA Western blot of MDA-MB-436 cells expressing BFP control, HA-BRCA1-full-length (FL), HA-BRCA1-ΔRING with aa start at M128 and M297. *Right*; mean and S.E.M. percentage foci positive cells; foci positive cells were assessed for the number of foci present in a single nucleus, red line: median value; foci positive cells were also assessed for the mean size of foci present per nuclei, n=3 biological replicates. Foci positive cells were determined to be those with ≥10 foci/nuclei. *** $p < 0.001$, ** $p < 0.01$, * $p < 0.05$, ^{ns} not significant compared to FL (percentage positive cells: unpaired, two-tailed t-tests size; number and size per nucleus: nonparametric Mann-Whitney tests). *Below*; representative images (scale bar, 10 μm). See Source Data for additional information.

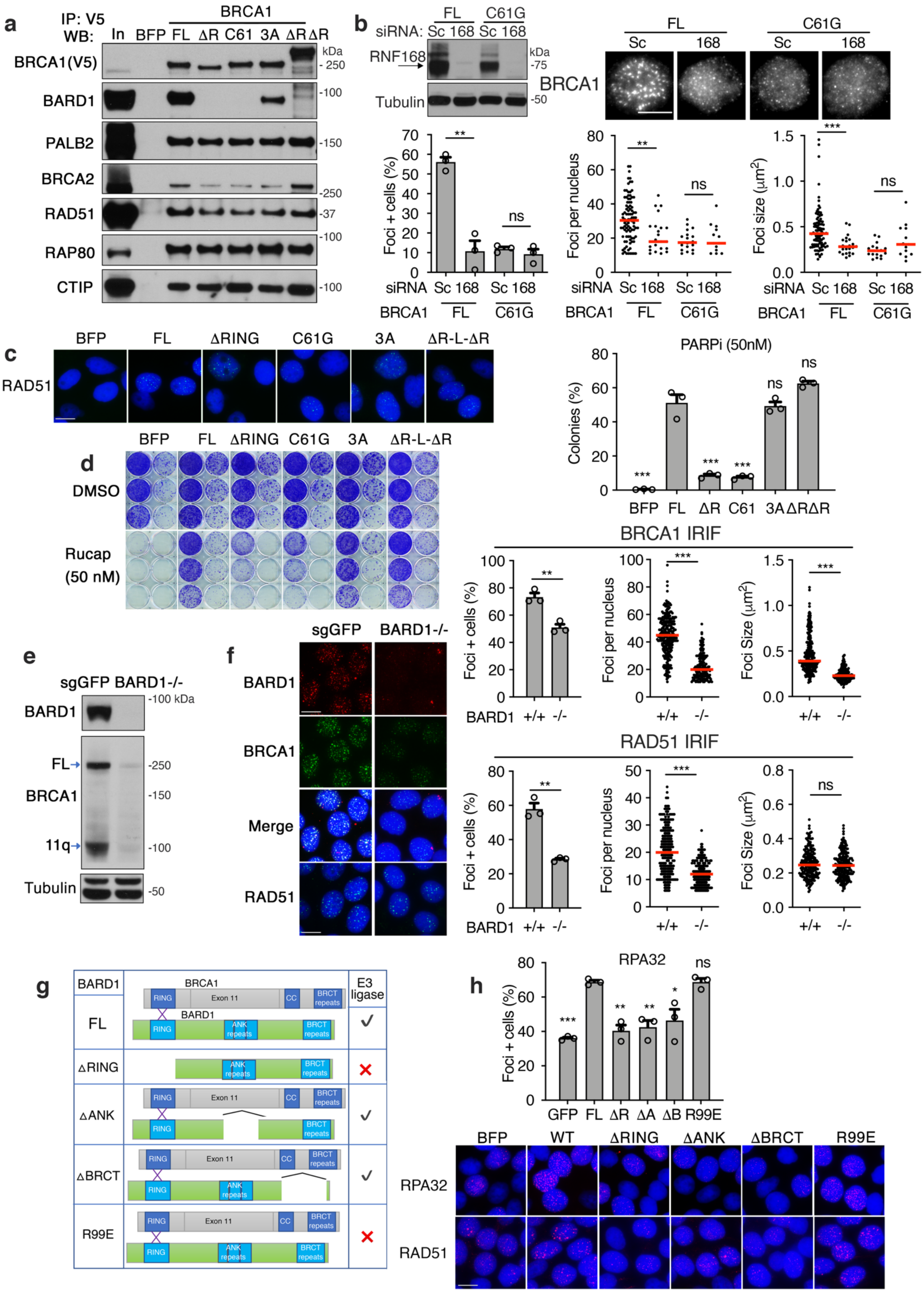

a
IP: V5
WB:
In BFP FL ΔR C61 3A ΔRΔR
BRCA1
BRCA1(V5)
BARD1
PALB2
BRCA2
RAD51
RAP80
CTIP
kDa
-250
-100
-150
-250
-37
-100
-100
b
FL C61G
siRNA: Sc 168 Sc 168
RNF168
Tubulin
kDa
-75
-50
BRCA1
FL C61G
Sc 168 Sc 168
Foci + cells (%)
Foci per nucleus
Foci size (μm²)
siRNA Sc 168 Sc 168
BRCA1 FL C61G
ns
c
BFP FL ΔRING C61G 3A ΔR-L-ΔR
RAD51
d
BFP FL ΔRING C61G 3A ΔR-L-ΔR
DMSO
Rucap (50 nM)
PARPi (50nM)
Colonies (%)
BFP FL ΔR C61 3A ΔRΔR
BRCA1 IRIF
Foci + cells (%)
Foci per nucleus
Foci Size (μm²)
BARD1 +/+ -/-
RAD51 IRIF
e
sgGFP BARD1-/-
BARD1
-100 kDa
FL→
BRCA1
11q→
-250
-150
-100
Tubulin
-50
f
sgGFP BARD1-/-
BARD1
BRCA1
Merge
RAD51
g
BARD1
E3 ligase
BRCA1
RING Exon 11 CC BRCT repeats
BARD1
RING ANK repeats BRCT repeats
FL
ΔRING
ΔANK
ΔBRCT
R99E
h
RPA32
Foci + cells (%)
GFP FL ΔR ΔA ΔB R99E
BFP WT ΔRING ΔANK ΔBRCT R99E
RPA32
RAD51

Supplementary Figure 3. Assessment of BRCA1 and BARD1 foci formation, related to Fig. 3.

(**A**) BRCA1 V5-tagged constructs expressed in MDA-MB-436 cells were immunoprecipitated and subject to Western blotting for the indicated proteins.

(**B**) MDA-MB-436 cells expressing BRCA1-FL or BRCA1-C61G were subject to scrambled (Sc) and RNF168-targeting siRNA followed by IR and HA foci formation as described in Fig. 2B. *Left*; Western blot showing RNF168 depletion. *Above*; representative cells (scale bar, 10 μm). n=3 biological replicates. See Source Data for additional information.

(**C**) Representative RAD51 foci images from Fig. 3C (scale bar, 10 μm).

(**D**) Representative 6-well plates showing colony formation of increasing cell densities that were seeded in the presence of either DMSO or 50 nM rucaparib. Mean and S.E.M. colonies that grew in the presence of rucaparib calculated as a percentage of those that grew in the presence of DMSO, n=3 biological replicates.

(**E**) MCF7 cells were subject to sgRNA targeting *GFP* or *BARD1*, individual colonies generated and *BARD1*$^{-/-}$ cells identified by Sanger sequencing. The effects of *BARD1* KO on BARD1, BRCA1 full-length and BRCA1-Δ11q protein expression were assessed by Western blotting.

(**F**) MCF7 cells harboring sgRNA targeting *GFP* and a *BARD1*$^{-/-}$ clone were assessed for BARD1, BRCA1 and RAD51 IRIF as described in Fig. 2A. *Left*; representative pictures (scale bar, 10 μm). *Right;* BRCA1 and RAD51 IRIF. Foci positive cells were determined to be those with ≥10 foci/nuclei for BRCA1 and ≥5 foci/nuclei for RAD51. See Source Data for additional information.

(**G**) Cartoon showing BARD1-full-length (FL), BARD1-ΔRING, BARD1-ΔANK, BARD1-ΔBRCT, and BARD1-R99E predicted effects on BARD1 interaction and E3 ligase activity used in Fig. 3E-H.

(**H**) Representative pictures as well as RPA32 IRIF mean and S.E.M. from cells treated as in Fig. 3G (scale bar, 10 μm), n=3 biological replicates. *** $p < 0.001$, ** $p < 0.01$, * $p < 0.05$, ^{ns} not significant (unpaired, two-tailed t-tests).

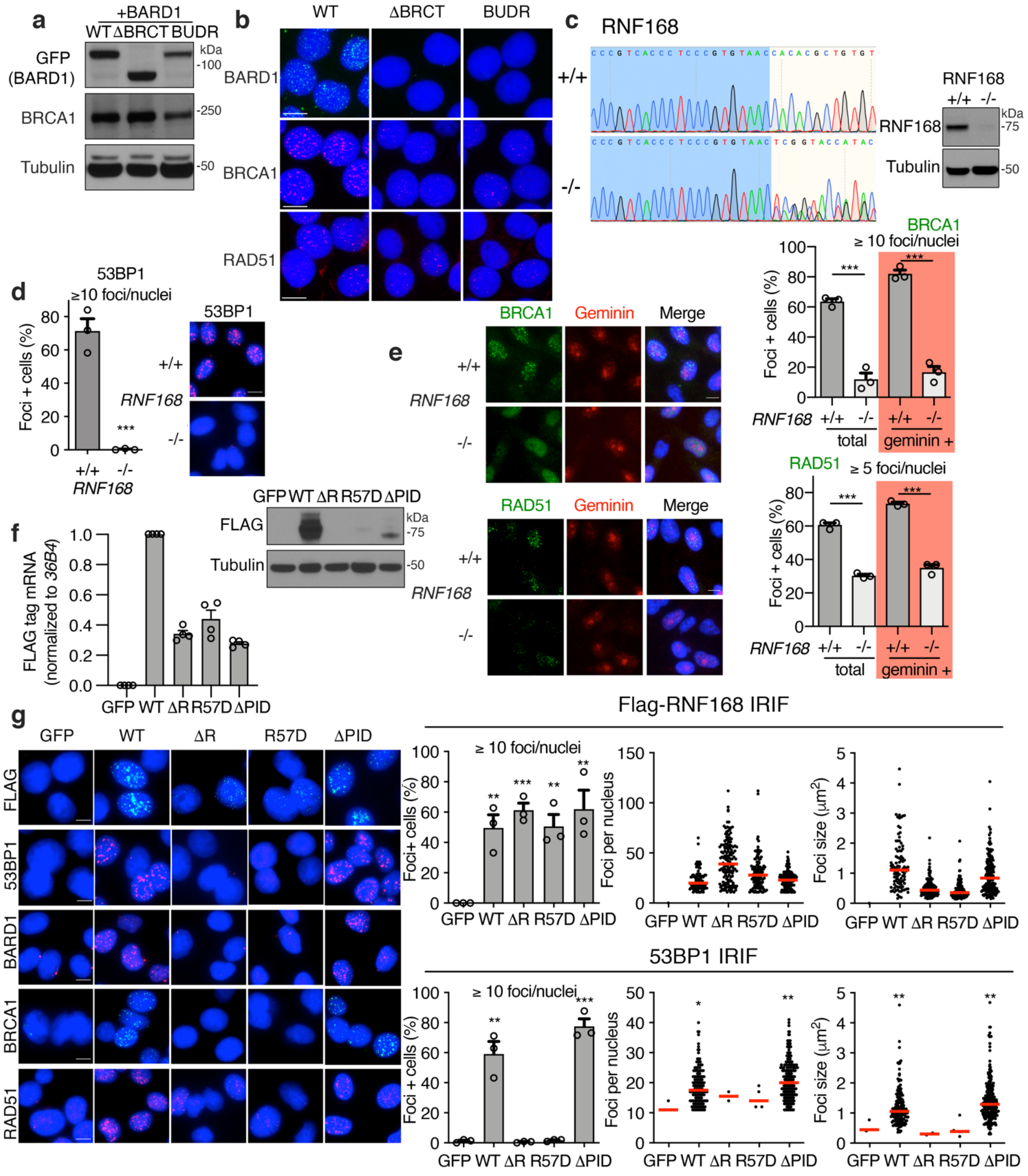

Supplementary Figure 4. Characterization of *RNF168* KO cells, related to Fig. 4.

(**A**) MCF7 *BARD1*$^{-/-}$ cells expressing GFP-BARD1-WT, GFP-BARD1-ΔBRCT, or GFP-BARD1-BUDR (R705A+D712A+Q715R) were assessed for GFP and BRCA1 expression by Western blotting.

(**B**) Representative foci images from Fig. 4A and B (scale bars, 10 μm).

(**C**) Electropherograms of *RNF168* DNA sequences from MDA-MB-231 WT and *RNF168*$^{-/-}$ cell lines are shown. The *RNF168*$^{-/-}$ cell line contains two alleles with frameshifting deletions. *Right*; Western blot showing loss of RNF168 expression.

(**D**) Cells from C were assessed for 53BP1 IRIF. Representative images of cells are shown. Mean and S.E.M. percentage foci positive cells are shown from n=3 biological replicates. *** $p < 0.001$ (unpaired, two-tailed t-tests).

(**E**) Cells from C were assessed for BRCA1 and geminin or RAD51 and geminin staining in the absence of pre-extraction by immunofluorescence. Representative images are shown. Mean and S.E.M. percentage foci positive cells as well as foci positive cells that were also geminin positive are shown from n=3 biological replicates. Geminin indicates S/G2 cell cycle fractions. *** $p < 0.001$, ** $p < 0.01$, * $p < 0.05$, ^{ns} not significant (unpaired, two-tailed t-tests).

(**F**) RNF168 ectopic UBC promoter constructs were assessed for Flag mRNA and protein expression by qRT-PCR (*left*) and Western blotting (*right*), respectively. Mean and S.E.M. are shown for n=4 technical replicates.

(**G**) Representative images from Fig. 4E as well as Flag-RNF168 and 53BP1 IRIF analyses as described in Fig. 4E for n=3 biological replicates. Scale bars, 10 μm for all panels. See Source Data for additional information.

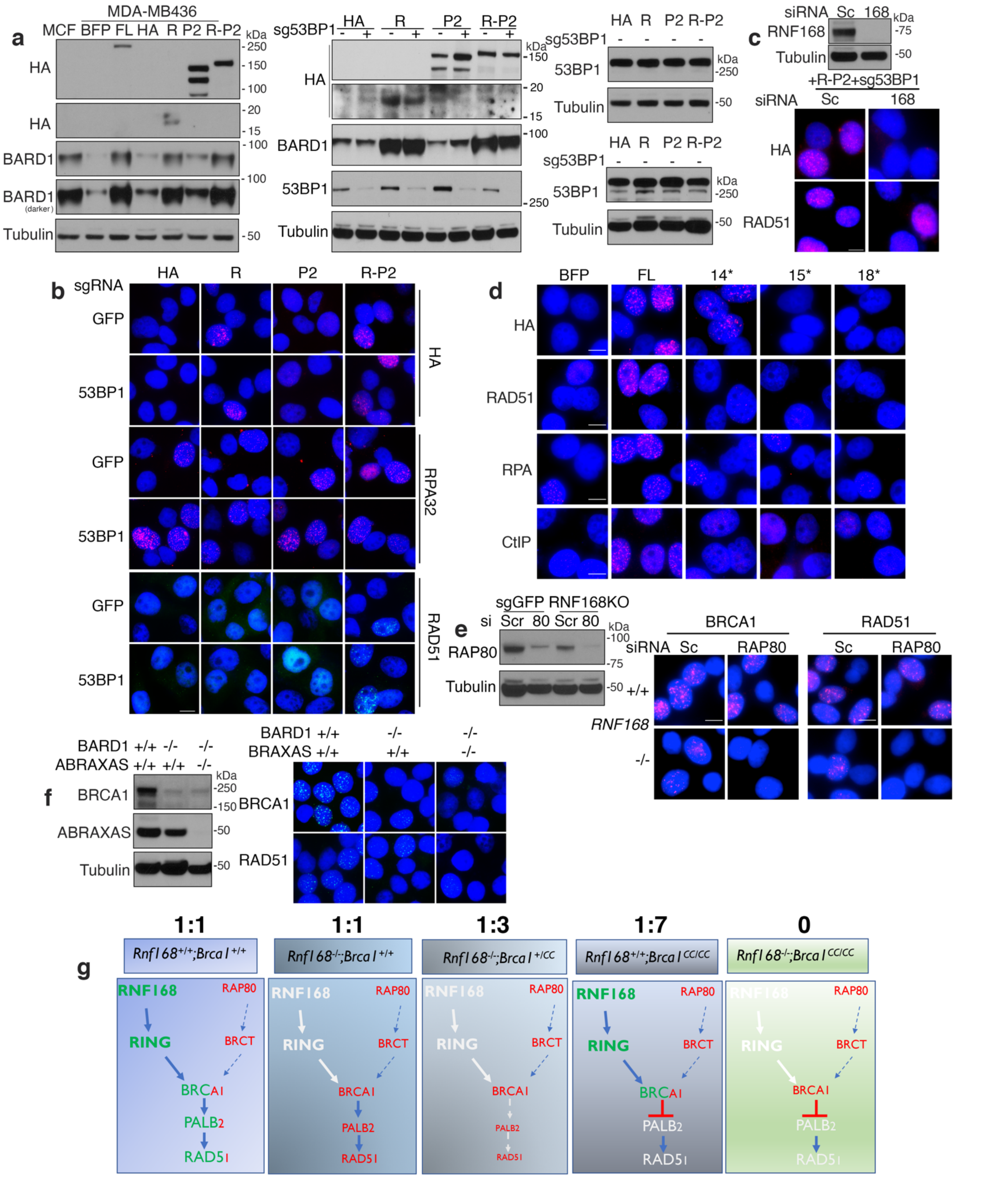

Supplementary Figure 5. RING constructs and the BRCA1-A complex, related to Fig. 4&5.

(**A**) Associated with Fig. 4G: *Left*, MDA-MB-436 cells expressing HA-empty vector (HA), HA-BRCA1 RING domain (R), HA-PALB2 (P2), or -HA-BRCA1 RING domain-PALB2 (R-P2) were subject to sgRNA targeting GFP or 53BP1 and assessed for the indicated protein expression by Western botting. To determine the relative

effects of RING constructs on BARD1 expression, protein levels were compared to MCF7 as well as MDA-MB-436 expressing ectopic BFP and full-length BRCA1. *Middle*, Western blots showing the effects of sg53BP1 on 53BP1 protein expression in the indicated cell lines. *Right*, Two sets of additional cell lysates were generated for 53BP1 wild-type cells to assess whether 53BP1 protein levels were similar between cell lines.

(**B**) Representative images from Fig. 4G.

(**C**) Representative images and western blot associated with Fig. 4I.

(**D**) Representative images associated with Fig. 5B.

(**E**) Representative images and western blot associated with Fig. 5E.

(**F**) Representative images and western blot associated with Fig. 5F.

(**G**) Model for impact of *Brca1*^{CC} and *Rnf168*$^{-}$ alleles and the supporting role of Rap80-Abraxas on Palb2 and Rad51 loading and development in mice. The Mendelian birth ratios of live mice are indicated above.

sgRNA DNA oligos	
m_sg168-F	caccAGAGACCCGGCGACGACAGA
m_sg168-R	aaacTCTGTCGTCGCCGGGTCTCT
m_rap80-F	caccATAGTGATATCCGATAGCGA
m_rap80-R	aaacTCGCTATCGGATATCACTAT
h_sg168-F	caccTCGAAAAGGCGAGTTTATGC
h_sg168-R	aaacGCATAAACTCGCCTTTTCGA
h_sgbard1-F	caccGCGACCATCCGGTTCCATGG
h_sgbard1-R	aaacCCATGGAACCGGATGGTCGC
h_sgabraxas-F	caccCCTCAACACGGACTCGGACA
h_sgabraxas-R	aaacTGTCCGAGTCCGTGTTGAGG
h_sg53bp1-F	caccGCCATCCAGTCCTCAAGGAG
h_sg53bp1-R	aaacCTCCTTGAGGACTGGATGGC
Sequencing Primers	
geno168com-F	GGGTCCTCACCGCGTAAGAAG
geno168wt-R	CGAAGAGACCCGGCGACGA
geno168del-R	GTATGGTACCGAGTCCATAAGGGACA
genoCC-F	GGTGCACTCTCCTCCAACATC
genoCC-R	CTCTTGCACCTGCCTCTCTGA
m_r80-F	GGTAAAAGGATGCCACGAAGGA
m_r80-R	CAAATAATATGACCACACGCGCT
h_168-F	TGATACGCTTCTGGGCATAATA
h_168-R	ACCCCCTAACCTCTGAGAACTAT
h_bard1-F	GTGCCCTGCGAGTCCCTAT
h_bard1-R	CAAAAACTACCGTTTCAGTTGGAT
h_abraxas-F	CAGCAGAAGCGAAGGAGGA
h_abraxas-R	GAGGGCTAATGCTGGAGAAGA
qRTPCR Primers	
flag-F	TACAAAGACCATGACGGTGATTA
flag-R	AGTCTCGCTGCCTGAGATA
HAB1-F	CCTACGACGTGCCCGACTA
HAB1-R	ATGGGACACTCTAAGATTTTCTGCA

Supplementary Table 1. List of primers
