# Supplementary material for: RNF168-mediated localization of BARD1 recruits the BRCA1-PALB2 complex to DNA damage
Source: Nat Commun. 2021 Aug 18;12:5016. doi: 10.1038/s41467-021-25346-4 (PMC8373961; doi:10.1038/s41467-021-25346-4)
Supplement: Supplementary file 2 — Reporting Summary [file 41467_2021_25346_MOESM2_ESM.pdf]

nature portfolio

Corresponding author(s): Neil Johnson

Last updated by author(s): Jul 22, 2021

Reporting Summary

Nature Portfolio wishes to improve the reproducibility of the work that we publish. This form provides structure for consistency and transparency in reporting. For further information on Nature Portfolio policies, see our Editorial Policies and the Editorial Policy Checklist.

Statistics

For all statistical analyses, confirm that the following items are present in the figure legend, table legend, main text, or Methods section.

n/a	Confirmed	
☐	☒	The exact sample size (*n*) for each experimental group/condition, given as a discrete number and unit of measurement
☐	☒	A statement on whether measurements were taken from distinct samples or whether the same sample was measured repeatedly
☐	☒	The statistical test(s) used AND whether they are one- or two-sided<br>*Only common tests should be described solely by name; describe more complex techniques in the Methods section.*
☒	☐	A description of all covariates tested
☐	☒	A description of any assumptions or corrections, such as tests of normality and adjustment for multiple comparisons
☐	☒	A full description of the statistical parameters including central tendency (e.g. means) or other basic estimates (e.g. regression coefficient) AND variation (e.g. standard deviation) or associated estimates of uncertainty (e.g. confidence intervals)
☐	☒	For null hypothesis testing, the test statistic (e.g. *F*, *t*, *r*) with confidence intervals, effect sizes, degrees of freedom and *P* value noted<br>*Give P values as exact values whenever suitable.*
☒	☐	For Bayesian analysis, information on the choice of priors and Markov chain Monte Carlo settings
☒	☐	For hierarchical and complex designs, identification of the appropriate level for tests and full reporting of outcomes
☒	☐	Estimates of effect sizes (e.g. Cohen's *d*, Pearson's *r*), indicating how they were calculated

Our web collection on statistics for biologists contains articles on many of the points above.

Software and code

Policy information about availability of computer code

Data collection	Image collection and projection images were generated by Nikon NIS Elements Br software version 4.60.00. Cell cycle profiles were collected with a BD Facscan.
Data analysis	Image analysis was performed using ImageJ (FIJI) version 2.1.0/1.53c. Statistical analysis was performed in RStudio 1.3.1093 or with GraphPad Prism 9. Cell cycle analysis was performed with FlowJo 10.7.1.

For manuscripts utilizing custom algorithms or software that are central to the research but not yet described in published literature, software must be made available to editors and reviewers. We strongly encourage code deposition in a community repository (e.g. GitHub). See the Nature Portfolio guidelines for submitting code & software for further information.

Data

Policy information about availability of data

All manuscripts must include a data availability statement. This statement should provide the following information, where applicable:

- Accession codes, unique identifiers, or web links for publicly available datasets
- A description of any restrictions on data availability
- For clinical datasets or third party data, please ensure that the statement adheres to our policy

All relevant data are available from the authors upon reasonable request. The data generated in this study are provided in the Source Data file. Guide RNA design was performed using https://portals.broadinstitute.org/gpp/public/analysis-tools/sgrna-design.

Field-specific reporting

Please select the one below that is the best fit for your research. If you are not sure, read the appropriate sections before making your selection.

☒ Life sciences ☐ Behavioural & social sciences ☐ Ecological, evolutionary & environmental sciences

For a reference copy of the document with all sections, see nature.com/documents/nr-reporting-summary-flat.pdf

Life sciences study design

All studies must disclose on these points even when the disclosure is negative.

Sample size	No statistical methods were used to pre-determine sample size. Most experiments were conducted with 3 independent replicates, unless otherwise indicated, selected based on standard practices.
Data exclusions	No data were excluded from the analyses.
Replication	Data was replicated successfully in 3 independent experiments, unless otherwise indicated. Number of replicates is provided in each figure legend.
Randomization	No randomization was performed.
Blinding	Non-biased image analysis was performed using ImageJ software.

Reporting for specific materials, systems and methods

We require information from authors about some types of materials, experimental systems and methods used in many studies. Here, indicate whether each material, system or method listed is relevant to your study. If you are not sure if a list item applies to your research, read the appropriate section before selecting a response.

Materials & experimental systems

n/a	Involved in the study	
☐	☒	Antibodies
☐	☒	Eukaryotic cell lines
☒	☐	Palaeontology and archaeology
☐	☒	Animals and other organisms
☒	☐	Human research participants
☒	☐	Clinical data
☒	☐	Dual use research of concern

Methods

n/a	Involved in the study	
☒	☐	ChIP-seq
☐	☒	Flow cytometry
☒	☐	MRI-based neuroimaging

Antibodies

Antibodies used

Immunofluorescence
Brca1 (gift from A. Nussenzweig),
BRCA1 (Millipore, 07-434),
RAD51 (Genetex, GTX100469),
RAD51 (Abcam, ab133534),
HA (Covance, MMS-101R),
BARD1 (Santa Cruz Biotechnology, sc-74559),
53BP1 (Novus Biologicals, NB100-305),
53BP1 (Millipore, MAB3802),
RPA32 (Cell Signaling, 2208),
RPA32 (Sigma-Aldrich, NA18),
RAP80 (Bethyl Laboratories, A300-763A),
FLAG (Sigma-Aldrich, F1804),
FLAG (Cell Signaling, 14793),
CtIP (Millipore, MABE1060),
Geminin (Abnova, H00051053-M01),

Western blot
HA (Cell Signaling, 2367),
RNF168 (R&D Systems, AF7217),
RNF168 (Millipore, ABE367),
BRCA1 (Millipore, 07-434),
BARD1 (Bethyl Laboratories, A300-263A),

GFP (Santa Cruz Biotechnologies,sc-9996),
FLAG (Sigma-Aldrich, F1804),
V5 (Bethyl Laboratories, A190-120A),
PALB2 (Bethyl Laboratories, A301-246A),
BRCA2 (Bethyl Laboratories, A303-434A),
RAD51 (Santa Cruz Biotechnology, sc-8349),
CtIP (Bethyl Laboratories, A300-488A),
RAP80 (Bethyl Laboratories, A300-763A),
ABRAXAS (Bethyl Laboratories, A302-180A),
53BP1 (Millipore, MAB3802),
RNF8 (Santa Cruz Biotechnology, sc-271462),
Tubulin (Cell Signaling, 2148)

Validation

Brca1 (gift from A. Nussenzweig) - we previously validated this antibody using MEFs derived from Brca1 mutant mice in Nacson et al. Cell Reports, 2018
BRCA1 (Millipore, 07-434) - validation stated at https://www.emdmillipore.com/US/en/product/Anti-BRCA1-Antibody,MM_NF-07-434
RAD51 (Genetex, GTX100469) - validation stated at https://www.genetex.com/Product/Detail/Rad51-antibody-N1C2/GTX100469
RAD51 (Abcam, ab133534) - validation stated at https://www.abcam.com/rad51-antibody-epr40303-ab133534.html
HA (Covance, MMS-101R) - validation stated at https://www.biolegend.com/en-us/products/anti-ha-11-epitope-tag-antibody-11071?GroupID=GROUP26
BARD1 (Santa Cruz Biotechnology, sc-74559)- validation stated at https://www.scbt.com/p/bard1-antibody-e-11
53BP1 (Novus Biologicals, NB100-305) - validation stated at https://www.novusbio.com/products/53bp1-antibody_nb100-305
53BP1 (Millipore, MAB3802) - validation stated at https://www.emdmillipore.com/US/en/product/Anti-53BP1-Antibody-clone-BP13,MM_NF-MAB3802
RPA32 (Cell Signaling, 2208) - validation stated at https://www.cellsignal.com/products/primary-antibodies/rpa32-rpa2-4e4-rat-mab/2208
RPA32 (Sigma-Aldrich, NA18) - validation stated at https://www.emdmillipore.com/US/en/product/Anti-Replication-Protein-A-Ab-2-Mouse-mAb-RPA34-19,EMD_BIO-NA18
RAP80 (Bethyl Laboratories, A300-763A) - validation stated at https://www.bethyl.com/product/A300-763A/RAP80+Antibody
FLAG (Sigma-Aldrich, F1804) - validation stated at https://www.sigmaaldrich.com/US/en/product/sigma/f1804
FLAG (Cell Signaling, 14793) - validation stated at https://www.cellsignal.com/products/primary-antibodies/dykddddk-tag-d6w5b-rabbit-mab-binds-to-same-epitope-as-sigma-s-anti-flag-m2-antibody/14793
CtIP (Millipore, MABE1060) - validation stated at https://www.emdmillipore.com/US/en/product/Anti-CtIP-Antibody-clone-14-1,MM_NF-MABE1060
Geminin (Abnova, H00051053-M01) - validation stated at http://www.abnova.com/products/products_detail.asp?catalog_id=H00051053-M01
HA (Cell Signaling, 2367) - validation stated at https://www.cellsignal.com/products/primary-antibodies/ha-tag-6e2-mouse-mab/2367
RNF168 (R&D Systems, AF7217) - validation stated at https://www.rndsystems.com/products/human-mouse-rnf168-antibody_af7217
RNF168 (Millipore, ABE367) - validation stated at https://www.emdmillipore.com/US/en/product/Anti-RNF168-Antibody,MM_NF-ABE367
BARD1 (Bethyl Laboratories, A300-263A) - validation stated at https://www.bethyl.com/product/A300-263A
GFP (Santa Cruz Biotechnologies, sc-9996) - validation stated at https://www.scbt.com/p/gfp-antibody-b-2
V5 (Bethyl Laboratories, A190-120A) - validation stated at https://www.bethyl.com/product/A190-120A
PALB2 (Bethyl Laboratories, A301-246A) - validation stated at https://www.bethyl.com/product/A301-246A
BRCA2 (Bethyl Laboratories, A303-434A) - validation stated at https://www.bethyl.com/product/A303-434A
RAD51 (Santa Cruz Biotechnology, sc-8349) - validation stated at https://www.scbt.com/p/rad51-antibody-h-92
CtIP (Bethyl Laboratories, A300-488A) - validation stated at https://www.bethyl.com/product/A300-488A
RAP80 (Bethyl Laboratories, A300-763A) - validation stated at https://www.bethyl.com/product/A300-763A
ABRAXAS (Bethyl Laboratories, A302-180A) - validation stated at https://www.bethyl.com/product/A302-180A
RNF8 (Santa Cruz Biotechnology, sc-271462) - validation stated at https://www.scbt.com/p/rnf8-antibody-b-2
Tubulin (Cell Signaling, 2148) - validation stated at https://www.cellsignal.com/products/primary-antibodies/a-b-tubulin-antibody/2148

Eukaryotic cell lines

Policy information about cell lines

Cell line source(s)

MDA-MB-436, MDA-MB-231 and MCF7 cell lines were obtained from ATCC. HEK293T were purchased from Takara Bio. Mouse embryonic fibroblast cell lines were generated.

Authentication

Cell line identities were confirmed by short tandem repeat (STR) profiling using IDEXX analysis.

Mycoplasma contamination

Cell lines were confirmed negative for mycoplasma contamination by Lonza MycoAlert assay LT07-705.

Commonly misidentified lines
(See ICLAC register)

No commonly misidentified lines were used in this study.

Animals and other organisms

Policy information about studies involving animals; ARRIVE guidelines recommended for reporting animal research

Laboratory animals	CRISPR editing of mouse embryos were performed in a B6C3F1/J background (offspring of C57BL/6J females and C3H/HeJ males from Jax Laboratory). Rnf168- mouse crosses were performed on a mixed background using males and females less than 6 months old; B6C3F1/J and C57BL/6J. Mice were house in standard conditions approved by the American Association for Accreditation of Laboratory Animal Care.
Wild animals	This study does not involve wild animals
Field-collected samples	This study does not involve field-collected samples.
Ethics oversight	The Fox Chase Cancer Center (FCCC) Institutional Animal Care and Use Committee (IACUC) approved experiments involving mice.

Note that full information on the approval of the study protocol must also be provided in the manuscript.

Flow Cytometry

Plots

Confirm that:

- [x] The axis labels state the marker and fluorochrome used (e.g. CD4-FITC).
- [x] The axis scales are clearly visible. Include numbers along axes only for bottom left plot of group (a 'group' is an analysis of identical markers).
- [ ] All plots are contour plots with outliers or pseudocolor plots.
- [x] A numerical value for number of cells or percentage (with statistics) is provided.

Methodology

Sample preparation	Exponentially growing cells were harvested and fixed with 50% ethanol at 4 degrees. Cells were washed with PBS and incubated in FxCycle PI/RNase staining solution (Thermo Fisher Scientific, F10797) for 30 min
Instrument	BD FACScan
Software	FlowJo 10.7.1
Cell population abundance	%G1, %S, %G2 phase cell populations are indicated for each cell line, 10000 events recorded per sample
Gating strategy	The PI histogram was used to gate %G1, %S, %G2 phase cell populations. representative histograms are in the figure.

- [x] Tick this box to confirm that a figure exemplifying the gating strategy is provided in the Supplementary Information.
